# Hospital and Institutional News

**Published:** 1913-05-17

**Authors:** 


### HOSPITAL AND INSTITUTIONAL NEWS.

### OUR HOSPITAL SUNDAY SPECIAL NUMBER.

This year a new departure occurs in our Hospital Sunday Special Number. Instead merely of publishing several articles by the clergy dealing with the question of the Fund or urging its claims, as was done last year, for instance, by Sir Thomas Boor Crosby, the then Lord Mayor, we have decided to deal with the attempt that has been made by anti-vivisectionists to hinder subscriptions. The recent decision in the libel action against the *Pall Mall Gazette* affords us an opportunity not to be wasted of showing that the general public can no longer be misled into withholding their subscriptions by the representations of anti-vivisectionists. In a striking article by Mr. Stephen Paget, F.R.C.S., the secretary of the Research Defence Society, the subject of "Hospital Sunday and Anti-Vivisection" is discussed and disposed of. Another article on "Wanton Enemies of Hospital Sunday," with one by the Bishop of North Queensland on "A Question of Religion," and another explaining "What London Hospitals Cost and Where the Money Comes From" conclude the literary matter of a Number, which by explaining the actual needs of the London institutions severally, should make the public realise the value of Hospital Sunday, and finally destroy the misrepresentations which have affected the total collected in the past. Any reader of The Hospital can obtain this Hospital Sunday Special, price one penny, from The Scientific Press, Limited, 28 and 29 Southampton Street, Strand, London, W.C.

### DEATH OF SIR EDMUND HAY CURRIE.

All who have learned to appreciate personal service in connection with the voluntary system will feel that a genuine loss has occurred to the hospitals, as well as to the Metropolitan Hospital Sunday Fund, by the death last Saturday of its secretary, Sir Edmund Hay Currie, at the age of seventy-nine. From 1872, the year of the Fund's foundation, he took the keenest personal interest in its welfare, and was one of its two honorary secretaries, Sir Richard Martin being the other. In 1902, after thirty years' work in this capacity, he became the paid secretary of the Fund. Though the Fund became the dearest interest of his life, and he used to say that his intimacy with the clergy gave a personal aspect to their co-operation, he was an active figure in other sides of institutional life. Educated at Harrow, he carried on his father's distillery at Bromley. Then he engaged in philanthropic work, and was chairman of the London Hospital for twelve years, being one of Mr. Holland's predecessors in that office. He was a member and at one time vice-chairman of the Metropolitan Asylums Board; chairman of the Trustees of the People's Palace for seven years since its foundation, and received a Knighthood in 1876, when Queen Victoria opened the building. He also founded the Seafield Technical College in Hampshire. Since the Fund was started, when Sir Sydney Waterlow was Lord Mayor, some two millions have been distributed to the hospitals and dispensaries in London. As a young man he went out to Scutari and the Crimea, and was fond of relating stories of the hospital conditions which prevailed. He made an attempt to enter politics by contesting the Tower Hamlets in 1874, but was unsuccessful, and one can hardly picture his gentle and courteous personality as suited to the rough and tumble of the party game. In an interview which he gave to our representative only a year ago in the Hospital Sunday Special Number, which appeared on June 9, 1912, on "The Growth and Future Prospects of the Metropolitan Hospital Sunday Fund," he laid down the principle on which he believed the future success of the Fund depended: "The clergy, the Sunday Fund, and the hospitals stand together . . . and so long as the Fund can maintain its intimacy, as a bridge and friend, between the clergy and the hospitals, so long will the Fund endure. Its maintenance, so far, has been a deeply personal one, individual enthusiasm and love for the work alone can maintain the necessary relation of my Fund to hospitals and clergy." This utterance is strongly characteristic of a man who always inspired affection, even in those who sometimes criticised the policy of the Sunday Fund.

### THE CRISIS AT SWANSEA.

The position at Swansea General Hospital which we referred to in our previous issue, owing to the action of the honorary staff in resolving to refuse to treat at the hospital those patients who belong to the medical-aid societies that have proposed to import practitioners from outside, became even more serious last week. The board of management has found it advisable to issue an appeal to the working-men subscribers, since a large number of letters, representing a still greater number of workmen, have been received stating that the usual annual subscriptions due this month will be withheld till a settlement be arrived at between the medical practitioners and the medical-aid societies. The board points out that the hospital, not through its own fault, has been placed in a position of grave difficulty. The hospital is in debt at present to the extent of £15,000, a sum which will soon be increased to £20,000 "if the resolution to hold up the panels is allowed to take effect." At the moment, too, the institution is being enlarged to meet the extended needs of the district, and the number of occupied beds is the highest in the history of the hospital. The stopping of subscriptions now would be disastrous, and the working men are asked to postpone any drastic action till the annual meeting takes place in July. Unfortunately it does not appear at the time of writing that this wise policy will be followed. The Landore and Swansea Valley Railwaymen's Hospital Fund have passed a resolution saying that they cannot pay their contribution at the present time. We can hope only that this example will not be followed, and that a conference may be held to settle by adjustment a dispute which by reprisals or "drastic action" falls hardly on innocent parties, whose interests must be the public's first care.

### NEW SCOTCH CHAIR OF BACTERIOLOGY.

Under a bequest from Mr. Robert Irvine, of Royston, Granton, a new Chair of Bacteriology, with a romantic story attached to its endowment, is to be founded at Edinburgh University. The *Challenger* Expedition found, thanks to Sir John Murray, an uninhabited island in the Indian Ocean which possessed valuable phosphates. Eventually Christmas Island, as it came to be called, was annexed by the British Government in 1888, and a company was formed to develop it. One of the shareholders was the late Mr. Robert Irvine, who bequeathed his interest to establish a Chair of Bacteriology in Edinburgh University when the interest from his shares should reach £30,000. The island has proved so productive that it has taken only eleven years, according to the *Geographical Journal*, to reach the stipulated sum. It is understood that £5,000 will be spent on laboratories, class-rooms, and their equipment, while the remainder will be spent on maintaining the professorship. It is also interesting to add that the remaining interest in the money received from the shares will pass ultimately in equal shares to the Edinburgh Royal Infirmary, the Royal Edinburgh Hospital for Incurables, and to the Dunlop Cancer Fund. It will be seen that Mr. Irvine was content to wait until the sum received from his investment was sufficient to form a liberal endowment for the chair he wished to found. We have often pointed out that it is as fatal as it is common to starve professorships if the best men and the best work are to be had. It is only poetic justice that, determining to wait for an adequate sum, fate has decreed that the time of waiting should be short.

### A CONGRATULATORY LETTER TO MR. ASTOR.

The unsuccessful suit which Miss Lind-af-Hageby lately brought against the *Pall Mall Gazette* involved the proprietor of that paper, Mr. Astor, in very heavy expense, notwithstanding that costs were given against the plaintiff. In recognition of Mr. Astor's public spirit in facing this expenditure rather than allow the cause of truth and progress to suffer, a congratulatory letter has been addressed to him, the signatures to which occupy two columns in the newspaper. The letter itself is brief and business-like, and shows unmistakably that every man who has signed it is a firm believer in vivisection as indispensable to the advance of the therapeutic sciences. The list of the signatories is well worth glancing through. We do not suggest that every distinguished surgeon, physician, or scientist in Britain has signed it; though probably every one of them with any reputation to lose would sign if he were asked to. But we do affirm that every signature to the letter is that of a man of standing and distinction, and that a large number are of the very highest character, eminence, and accomplishments. When men of such unblemished reputation and acknowledged talents are found to be unanimous over a matter in which they are one and all experts, it is easy to see why juries refuse to adopt the views of a small but strident section of would-be reformers.

### A REASONABLE LIMIT FOR PANEL DOCTORS.

We are glad to see that a meeting was held last week of panel and non-panel doctors in the City, presided over by Sir Thomas Boor Crosby, at which the allocation of several thousands of people to men on the panel was stigmatised as making "honest and just treatment" impossible. The resolution embodying this phrase was moved by Dr. Gillies, and concluded with the following demand: "We therefore ask that a reasonable limit be put on the number in the care of any one medical man." Dr. Scanlan remarked that numbers alone did not make medical service impossible: it depended on the ages of the patients as well. For example, he said that it would be quite easy to have "some thousands of patients between the ages of twenty and thirty," while "between the ages of forty and sixty it would be quite another matter." As an army doctor he had several thousands of men under his care. It was necessary to group patients. That is all very well, but Dr. Scanlan must remember that insured persons are still supposed to have free choice not of doctors, but at least of "panelites," and that in order to group patients in his system the doctors or other authorities would have to decide on the patients, instead of the patients deciding on their doctors. To carry out his system would mean, we fear, depriving the unfortunate insured class of the last tatters and shreds of liberty left to them. Even apart from this, it would increase the already great administrative difficulties to allocate groups of patients according to their age.

### AN ACCUSED'S OBJECTIONS TO DOCTOR'S FEES.

At the North London Police Court last Monday a man named John Sayers was charged with being drunk and disorderly. On the magistrate imposing a fine of three shillings and sixpence, and ordering him to pay a similar amount as the doctor's fee, for a medical man had been called in to see him at the station, Sayers objected. "Under Mr. Lloyd George's Insurance Act," he said, "I have medical attendance free. I have paid my insurance right up to date, and I told the police to call my panel doctor." The magistrate said that he did not think that it was part of the duty of a panel doctor to attend an insured person under such conditions, and the man must pay the fee. Many people will question this judgment, for the moment a distinction is attempted between illnesses caused "naturally" and those the result of personal indiscretion it is hard to know where to stop. The worst of sick insurance is that the interests of the insured person and of those who control the bestowal of benefits are opposed. When a man is compelled to insure, this opposition comes home to him with the greater bitterness.

### THE INSURED ON INSURANCE COMMITTEES.

On July 15, in theory at any rate, the representatives of the insured will begin to hold office for three years on the Insurance Committees. The regulations for the election of such representatives were issued last week by the Insurance Commissioners. Societies, we learn, having a membership equal to the unit of representation for their area will be entitled to appoint one or more representatives directly. The remaining seats will be filled, by election, by the members of the smaller societies, the system employed being that of the single transferable vote. Deposit contributors are also to be represented. Applications for nomination forms will be received up to noon on May 27 and the nominations must be made by noon on June 9. Ballot papers will be issued to societies on June 20 and will be returnable by June 27. It is perhaps not unnecessary to repeat that the interests of insured persons are not necessarily the same as those of approved societies, and we are inclined to doubt whether the former will gain much or at all from the suggested representation.

### DUAL CONTROL AND SANATORIUM BENEFIT.

It has more than once been noticed in these columns how much overlapping of effort in respect of providing for consumptive patients was already being carried on before the dubious sanatorium benefit was added to increase the difficulties of satisfactory provision. Now Dr. H. Muir Evans has written to the lay press pointing out how the County Councils are forwarding to the Local Government Board schemes for institutional treatment, dispensaries, and sanatoria without consultation with the Insurance Committees, which are responsible for providing for the treatment of insured persons. He then gives two examples of the way in which the minds of the medical experts on the Insurance Committees are being driven to despair. "County Councils," writes Dr. Muir Evans, "are proposing to appoint their medical officer of health as tuberculosis officer, although his duties should be only that of an administrative head." We agree that it is asking too much to expect one man to be an expert in both preventive medicine and treatment. Also it is clearly undesirable for the medical officer of health to delegate his consultative duties to assistant tuberculosis officers, whose small experience is indicated by the fact that the salaries offered are only £300 a year. Then Dr. Muir Evans shows that, while "sanatoria can be erected at so small a sum as £60 per bed," it is proposed in some areas to build hospitals for the incurables and asylums for the dying, whose presence is a menace to their dependants, at a cost of £150 per bed, apart from the cost of site. "Officials," he concludes, "always prefer expenditure on bricks and mortar rather than on men and brains." Dr. Evans would add to the value of his criticisms if he would formulate a scheme for co-ordinating the Insurance Committees' responsibilities with the activities of the County Councils. We invite him to develop this aspect of a difficult question.

### FROM MANOR HOUSE TO SANATORIUM.

Mr. D. W. Evans, general director of the King Edward Welsh National Memorial, purchased last week at a sale at Brecon the Pontywall Hall Estate and House for £15,600. The estate is really a picturesque one, and the house stands six hundred feet above the level of the sea, commanding a fine view. Mr. Evans announced that the house would be used for a sanatorium for the treatment of consumptive patients in the two counties of Breconshire and Radnorshire. This may be regarded as one of the most important new institutions which has been mentioned in connection with the Welsh Memorial scheme. The story of its adaptation will be instructive, for the opinion is gaining weight that a sanatorium needs as much specialised design as a hospital.

### THE DUCHESS OF CONNAUGHT.

After the satisfactory progress disclosed by the bulletins since the first operation performed by Mr. Lane upon H.R.H. the Duchess of Connaught the public was distinctly disappointed by the announcement that there has been a recurrence of symptoms of intestinal obstruction, and that a further operation had been necessitated. In Mr. Arbuthnot Lane the Duchess has a surgeon who commands the confidence not only of the public, but also of his colleagues in the profession, and in expressing the hope that this second operation will restore her to complete health, it can be added with truth that, if this happy result is humanly possible, Mr. Lane is the man to accomplish it. At the moment of going to press it is obvious from the steadily diminishing reserve of the official bulletins that her Royal Highness is practically out of all immediate danger, and making progress which if necessarily slow is at least uninterrupted. We are but echoing the universal sentiment of the nation at large in recording the hope that the rest of her convalescence may be speedy, safe, and certain.

### THE UTILISATION OF DAY ROOMS.

What would hospital authorities have thought a few years ago of the suggestion made by Dr. W. G. Nash that the day rooms at the Bedford County Hospital should be used to reduce the waiting list? What would they have thought of his further statement that "the only solution was to utilise the day rooms outside each ward, which had never been used by the patients for whom they were intended, but at the present time are used as far as I know for storage purposes and for the sisters to have tea in? They are really wasted." He felt very strongly, and the medical committee recommended that these rooms should have three or four beds put into them, and so increase the number of surgical beds. The fact appears to be that the modern tendency of reducing the stay of patients in the wards to the lowest possible time has had the effect of removing patients before they reach the convalescent stage, with the result that the day room has largely ceased to have its former function. Indeed, when the position of the day rooms in some of the older institutions is considered, it appears that this room has given way to the modern balcony, which nowadays is often covered in on three sides, as when placed between two sanitary towers, for instance. No more vivid comment on the tendency to reduce the length of stay in the wards can be found than this instance of day rooms not used for their original purpose, and now claimed for surgical beds.

### WORCESTER INFIRMARY AND SICKNESS BENEFIT.

In view of the fact that a sub-committee has been appointed to consider what steps shall be taken to bring the expenditure of the Worcester General Infirmary into line with its income it is interesting to note that at the monthly meeting the secretary reported on the negotiations with the different Insurance Societies, and stated that all but two had paid them the sickness benefit due in the case of those patients who had no dependants. At the present rate of payment, the infirmary would receive about £200 per annum. In reference to the Bishop's appeal, the Chairman said that so far the response had been disappointing. £77 in donations and £72 19s. in new subscriptions had been received. As the number of hospital patients without dependants is generally small the sum of £200 per annum appears to be rather surprising as an estimate of income from this source.

### THE ASYLUM WORKERS' ASSOCIATION.

The Annual General Meeting of the Asylum Workers' Association will be held at 11 Chandos Street, Cavendish Square, on Wednesday, May 28, when the chair will be taken at three o'clock by Sir John Jardine, K.C.I.E., the President of the Association. Dr. G. E. Shuttleworth is the honorary secretary. In addition to the election of officers and to the submission of the annual report, presentations of medals will be made for long service.

### THE ANALYSIS OF SECRET REMEDIES.

Mr. E. F. Harrison, the analyst responsible for most of the analyses published in "Secret Remedies," has made a full reply to the witnesses before the Select Committee on Proprietary Medicines who had criticised that book adversely. With regard to Steedman's Powders, the powders in one packet were found by him to vary in weight from 1.9 to 4.5 grains, pointing to extreme carelessness, and it was not unreasonable to suppose that there may have been some carelessness in mixing and that the powders examined by him contained even less of one of the ingredients than the quantity intended to be present. It did not follow that if another analyst found a certain ingredient in a sample of medicine supplied by the proprietors or purchased in the ordinary way the same ingredient was in a sample which he had analysed from one to six years previously. Sir Joseph Beecham and Mr. South (of Steedman's) had admitted having altered their formulæ, while Powell's Balsam and Winslow's Syrup also used to contain morphine, but did not now contain it. But, apart from deliberate alterations, there were variations, due apparently to careless mixing. With regard to Woodward's Gripe Water, he was prepared to admit that it contained a trace of pungent aromatic substance; one of the witnesses called this "the most important ingredient." Yet other witnesses had expressed the opinion that another infants' medicine was practically unaffected by the presence or absence of a trace of a far more potent drug—opium. "That well illustrates," added Mr. Harrison, "that so long as a proprietor can keep a medicinal ingredient secret he can make very great claims for its medicinal virtues."

### DR. ADRIAN WILSON'S WILL.

All interested in the unhappy, if heroic, Antarctic expedition will not fail to note the proving of the will of the late Dr. Edward Adrian Wilson, chief of the staff to the Scott Antarctic Expedition, who perished with several other members of the party on their way back from the South Pole in March of last year. The estate is sworn by one of Dr. Wilson's brothers at £965 gross and net. The will, which was made nearly twelve years ago—that is to say, about the time of his marriage—leaves all the household furniture, plate, linen, books, pictures, and drawings to his wife, Mrs. Oriana Wilson, and all the other estate to executors on trust to pay the income thereof to his wife for life. This is but another reminder to the public of the need that exists for making suitable provision for the direct dependants of those who lost their lives to satisfy human curiosity—the divine impulse to which all scientific and other knowledge is due.

### DIFFICULTIES OVER THE WESTMINSTER SITE.

As we foresaw, æsthetic considerations are beginning to create heart searchings and consequent difficulties over the two sites of Westminster Hospital, the historic one which it still occupies, and the one which it is suggested it may occupy by Clapham Common. The protection of Broad Sanctuary was discussed at the meeting of the Westminster City Council last week, when, as a matter of urgency, Councillor R. W. Granville Smith said that he understood that the London County Council and the hospital authorities were coming to an agreement with the result that the future owner of the site would be able to bring a building over the existing porch, which would be a serious encroachment on Broad Sanctuary, and that the building might be 80 feet high. He moved that steps be taken to prevent any such agreement from being arrived at without the knowledge and consent of the City Council. It was decided to approach the Office of Works and the London County Council on these lines. The present position is that the private Bill promoted by the hospital authorities, by which power is sought to dispose of the present site and to acquire a new one in the County of London, has passed the second reading. The hospital authorities are also discussing with the London County Council the insertion of amendments at the Committee stage for a rearrangement of the frontage level to enlarge Broad Sanctuary.

### THE NEW SITE FOR WESTMINSTER HOSPITAL.

Hard upon the agitation in and out of Parliament concerning the proper utilisation of the Broad Sanctuary site where the Westminster Hospital at present stands, comes a loud protest as to the disposal of certain buildings on the new site at Clapham to which the hospital is expected to be moved. Clapham at present consists partly of mid-Victorian gloomy, stuccoed houses, and more largely still of late-Victorian jerry-built villas of the doll's house type. But at one corner of the north side of the Common there still survive some really picturesque relics of early eighteenth century architecture, which form a charming contrast to the drab dinginess of all the rest of the neighbourhood. It is feared that the removal of Westminster Hospital to Clapham will involve the destruction of this one oasis in the desert; and a strong protest is accordingly entered against the scheme. We are sufficiently in sympathy with the preservation of any really artistic remains of Old London to hope that some way out of the difficulty will be found.

### THE FEAR OF PATRONAGE.

Guardians, and other bodies with the power of selecting officials, are generally supposed to keep the right to make appointments as a jealous possession. This is generally the case, so generally that an instance to the contrary is worth recording. The managers of the Poplar and Stepney Sick Asylum have decided to ask the Local Government Board to make the appointment of their new clerk, "knowing," in the words of Father Higley, "they could not trust themselves" to do so. The names of six candidates, selected from forty, have therefore been forwarded to the Local Government Board. Humour is all very well in its place, and we should be the last to object to its timidly showing its head in the conduct of institutional affairs, but in this case we fear that managers who cannot trust themselves to appoint a clerk cannot be held worthy of any heavier responsibility.

### CARDIFF GUARDIANS AND A MEDICAL OFFICER.

It is generally recognised that there is a difficulty in securing medical officers for voluntary hospitals, but the Cardiff Board of Guardians have recently received only one applicant for the post of medical officer to the central district. The position is worth, including small fees for boarded-out children and isolation "pauper" mental patients, £200 a year. The matter was eventually referred to the general purposes committee. The question again was raised as to the desirability of appointing a whole-time officer rather than one engaged in private practice. It was suggested also that there was only one candidate because the applicant knew that he would be the only one. If this means that he and every other possible candidate thought the appointment would be bound to go to one man they were mistaken. The Guardians apparently could not face the position of appointing the only candidate they had. If a whole-time officer be required, a larger salary will probably become essential.

### SCHOOL DOCTORS ON MEDICAL INSPECTION.

The recent conference of Scottish school medical officers, which took place at Edinburgh, among the differences which it revealed in matters of detail, brought to light two main currents of opinion as to the best way in which to remedy the physical defects of school children. These contending views may perhaps be summarised in two questions. Should the school be made the centre of medical inspection, or the home? The majority of speakers appeared to be in favour of the former, but there was a minority which appeared to think that the school is not the proper place for such an undertaking. It is, of course, obvious that the doctor may supplement but cannot replace the school teacher; that, in short, the school can never became a hospital, but the tendency has everywhere been to show that where a public building is not made the scene of treatment the difficulties in the way of carrying that out become, in the end, overwhelming. Attach a clinic to the school, and though it may only be a beginning, it is a beginning that home inspection could never replace.

### THE INSPECTION OF RAILWAY CARRIAGES.

The suggestion again has been made that the railway companies should appoint sanitary officers and medical inspectors. There is no doubt that the general condition of railway carriages and platform or train lavatories is cleaner and more wholesome than used to be the case, but this seems due rather to the fact that the standard of comfort has risen, and with it the standard of cleanliness, than to any direct care in the matter itself. It is still common to find a scarcity of water for flushing purposes in train lavatories, and the dropping of a book on a cushioned seat is often quite sufficient to disturb a cloud of microbe-laden dust. The moral from what has been achieved so far appears to be that when money has once been spent on making the surroundings more comfortable objectionable habits become shamed into disuse. Spitting, for instance, undoubtedly receives a check in carriages or waiting rooms with a carpeted floor. This much is to the good, but the way to level up consistently and all the way round would appear, here as elsewhere, to provide adequate inspection. Dr. W. H. Whitehouse, medical officer for Deptford, has recently been driving this home.

### A MOTOR AMBULANCE FOR LONDON.

The London County Council has at last taken a definite step in the direction of improving the ambulance service of London by agreeing to contribute £300 a year towards the maintenance of a motor ambulance which the Grand Duke Michael has presented to the Hampstead General and North-West London Hospital, of which he is president. The hospital authorities are prepared to maintain the ambulance equipped for immediate service and to provide an attendant in addition to the driver. The ambulance is to be utilised for street accident cases, and it will serve the districts of Hampstead, St. Pancras, and the northern part of St. Marylebone. It will be stationed either at the hospital on Haverstock Hill or at the out-patient department at Camden Town, and is to be delivered to the hospital shortly.

### A JURY AND MISTAKEN DIAGNOSIS.

Juries sometimes show as much wisdom in refraining from adding a rider as in drawing one up. An inquest in the Southwark Coroner's Court last Saturday provides an instance. A man, described by a lad as having been "knocked out," was taken to Guy's Hospital, where he was detained and then ordered home by one of the staff. Eventually he became worse, the police surgeon was called in and sent him back to the hospital with a note as follows: "Query: Depressed fracture of the skull?" The police ambulance brought the man back to Guy's, where he was examined and ordered to the workhouse, where death took place. It was also stated that the man was brought to the workhouse on a lunacy order, and an autopsy revealed a fracture of the skull. A mistaken diagnosis was therefore admitted, though the man was seen by five medical men. Dr. Waldo, the City Coroner, in summing up, said that he had no doubt that Guy's Hospital had acted to the best of their ability in an extremely difficult case. The jury returned a verdict of accidental death, due to a fall, and said that they had no rider to add.

### RETIREMENT OF DR. E. I. SPRIGGS.

Not only St. George's Hospital and School, but the whole cause of medical education in London, is poorer by the retirement of Dr. E. I. Spriggs from his position as assistant physician at St. George's Hospital, where he was also for a long time Dean of the School. Some months ago Dr. Spriggs' health broke down seriously, and he was granted leave of absence by the governors in consequence. Until lately it was hoped that he would be able to resume his hospital work and his practice, but a decision has recently been arrived at that this would entail too great a risk, and Dr. Spriggs has accordingly resigned his post. He was appointed to it rather more than eight years ago, and was next on turn for promotion to the in-patient staff, which makes the enforced resignation particularly hard lines. Dr. Spriggs was educated at Guy's Hospital, but he identified himself so fully with the interests of his adopted hospital that he was soon chosen to be Dean, in which capacity he bore a prominent part in the discussions on the medical faculty of the University of London and the various schemes of reform. His many friends will wish him complete restoration to health in Scotland, where he is to take over the control of a new institution wholly devoted to the accurate diagnosis and modern treatment of disorders of metabolism. Dr. Spriggs is already one of the leading authorities in Britain on this subject, and the success of this well-considered project would seem to be assured.

### PASTORAL VISITATION IN HOSPITALS.

There is probably no question in the whole administration of a modern hospital which is more dependent on local "exigencies" than the position and duties of a hospital chaplain. In some places the jealousy of the different sects is so great that no chaplain is appointed, and it is merely left to patients and their friends to secure the visitation that they need. In Poor-Law infirmaries, as at the Firvale Infirmary, Sheffield, it was recently urged that the chaplain was one of the officers whom the Guardians were authorised to appoint, though they had preferred hitherto to rely on voluntary agencies. Again, it is local conditions, including the size of the hospital, which decide whether the chaplain should be whole-time officer or not. Such being the case, it is perhaps surprising that more complaints are not made than actually occur.

### QUEEN'S HOSPITAL FOR CHILDREN.

The latest report of the Queen's Hospital for Children, Hackney Road, reveals a serious position of affairs, and we understand that advantage will be taken of the annual meeting on the 19th instant to explain the present difficulties and to inaugurate an appeal. Subscribers must not be misled by the fact that the receipts for general purposes slightly exceeded the expenditure, for this result was only achieved at the cost of utilising all the legacies, which amounted to nearly £3,000, for the maintenance of the institution. To the total debt, including the mortgage of £2,510, must therefore be added the sum received from legacies last year, for such extraordinary income is a very fluctuating quantity, and even were it less so than it is, cannot permanently be used as ordinary income if the hospital's financial position is to be secured on a proper and healthy basis. The appeal which will be issued must therefore regard the £5,000 as the amount of the existing debt, and it is only when that sum has been subscribed that a beginning can be made in putting aside sums for future use and impending emergencies. That the latter are imminent as well as serious will be recalled by those who realise that the scheme of re-building is not complete, and that the commencement of a new out-patient department is earnestly hoped for by the chairman, Colonel Lord William Cecil, and the secretary, Mr. T. Glenton-Kerr. Sir Herbert Beerbohm Tree is to take the chair at the approaching meeting, and we hope that he will give the appeal a good send-off.

### THIS WEEK'S DRUG MARKET.

Owing to the intervention of the Whitsuntide holidays business has been very quiet, and few transactions of any importance have taken place, and few changes in value. Somewhat contrary to anticipation the price of cocaine is inclined in an upward direction; this is due to a rise in the price of coca leaves. The Opium market is quiet and the position unchanged. Menthol is slightly cheaper. Cream of tartar has a dearer tendency, as also has citric acid. Saffron is dearer.

